# Awareness of India’s national health insurance scheme (PM-JAY): a cross-sectional study across six states

**DOI:** 10.1093/heapol/czac106

**Published:** 2022-12-07

**Authors:** Diletta Parisi, Swati Srivastava, Divya Parmar, Christoph Strupat, Stephan Brenner, Caitlin Walsh, Rupak Neogi, Sharmishtha Basu, Susanne Ziegler, Nishant Jain, Manuela De Allegri

**Affiliations:** Heidelberg Institute of Global Health, Medical Faculty and University Hospital, Heidelberg University, Im Neuenheimer Feld 130.3, Heidelberg 69120, Germany; Heidelberg Institute of Global Health, Medical Faculty and University Hospital, Heidelberg University, Im Neuenheimer Feld 130.3, Heidelberg 69120, Germany; King’s Centre for Global Health and Health Partnerships, King’s College London, Strand, London WC2R 2LS, UK; German Institute of Development and Sustainability, Tulpenfeld, Bonn 6 53113, Germany; Heidelberg Institute of Global Health, Medical Faculty and University Hospital, Heidelberg University, Im Neuenheimer Feld 130.3, Heidelberg 69120, Germany; Heidelberg Institute of Global Health, Medical Faculty and University Hospital, Heidelberg University, Im Neuenheimer Feld 130.3, Heidelberg 69120, Germany; Nielsen India Private Limited, 7th floor Infotech Center 404-405 Near Country Inns and Suites, Phase III, Gurugram 122016, India; Indo-German Social Security Programme (IGSSP), Deutsche Gesellschaft für Internationale Zusammenarbeit (GIZ) GmbH, B – 5/1 & 5/2 Ground Floor, Safdurjung Enclave, New Delhi 110029, India; Heidelberg Institute of Global Health, Medical Faculty and University Hospital, Heidelberg University, Im Neuenheimer Feld 130.3, Heidelberg 69120, Germany; Deutsche Gesellschaft für Internationale Zusammenarbeit (GIZ) GmbH, Friedrich-Ebert-Allee 32+36, Bonn 53113, Germany; Indo-German Social Security Programme (IGSSP), Deutsche Gesellschaft für Internationale Zusammenarbeit (GIZ) GmbH, B – 5/1 & 5/2 Ground Floor, Safdurjung Enclave, New Delhi 110029, India; Heidelberg Institute of Global Health, Medical Faculty and University Hospital, Heidelberg University, Im Neuenheimer Feld 130.3, Heidelberg 69120, Germany

**Keywords:** Health insurance, awareness, India, social protection

## Abstract

The literature suggests that a first barrier towards accessing benefits of health insurance in low- and middle-income countries is lack of awareness of one’s benefits. Yet, across settings and emerging schemes, limited scientific evidence is available on levels of awareness and their determinants. To fill this gap, we assessed socio-demographic and economic determinants of beneficiaries’ awareness of the Pradhan Mantri Jan Arogya Yojana (PM-JAY), the national health insurance scheme launched in India in 2018, and their awareness of own eligibility. We relied on cross-sectional household (HH) survey data collected in six Indian states between 2019 and 2020. Representative data of HHs eligible for PM-JAY from 11 618 respondents (an adult representative from each surveyed HH) were used. We used descriptive statistics and multivariable logistic regression models to explore the association between awareness of PM-JAY and of one’s own eligibility and socio-economic and demographic characteristics. About 62% of respondents were aware of PM-JAY, and among the aware, 78% knew that they were eligible for the scheme. Regression analysis confirmed that older respondents with a higher educational level and salaried jobs were more likely to know about PM-JAY. Awareness was lower among respondents from Meghalaya and Tamil Nadu. Respondents from Other Backward Classes, of wealthier socio-economic status or from Meghalaya or Gujarat were more likely to be aware of their eligibility status. Respondents from Chhattisgarh were less likely to know about their eligibility. Our study confirms that while more than half of the eligible population was aware of PM-JAY, considerable efforts are needed to achieve universal awareness. Socio-economic gradients confirm that the more marginalized are still less aware. We recommend implementing tailored, state-specific information dissemination approaches focusing on knowledge of specific scheme features to empower beneficiaries to demand their entitled services.

**Key messages**

- Implications for policymakers
- While a considerable proportion of eligible beneficiaries know about PM-JAY, more needs to be done to increase both general awareness and depth of knowledge of specific scheme features.
- State-specific differences in general awareness and awareness of one’s eligibility of PM-JAY suggest that the legacy of past health insurance schemes may be affecting the current awareness and knowledge of eligible beneficiaries and that tailored, state-specific information, education and communication approaches are needed.

- Implications for public
- This study provides the first large-scale evidence on individual and HH determinants of awareness of PM-JAY and of one’s own eligibility. We find evidence of sex-, caste- and educational attainment–related gradients in awareness. State-specific differences in awareness and knowledge levels among eligible beneficiaries were also observed. Tailored, state-specific information dissemination approaches are needed focusing not only on general awareness but also on specific knowledge of scheme features so that beneficiaries can be empowered to demand their entitled services.

## Introduction

Universal Health Coverage (UHC) sits at the core of the Sustainable Development Goals (SDGs), being one of the specific targets embedded within SDG3 (Kieny *et al.*, 2017). The concept of UHC refers to people having access to basic quality health services without facing financial hardship. Moreover, the United Nations pledge ‘Leaving No One Behind’ is embedded within the concept of UHC, since the word universal assumes that all people, irrespective of socio-economic status, should enjoy a right to social health protection (World Health Organization, 2017). The concept implies that explicit efforts ought to be made to reach out to the most vulnerable to compensate for existing inequities in access to services and social health protection (Sidibé, 2016).

Given its central role within the SDGs, countries across the globe are implementing a number of reforms aimed at achieving UHC. In particular, countries are reforming their health financing structures to ensure that direct payments at the point of use are replaced by prepayment and pooling mechanisms, as these represent an essential condition to ensure access to services and social health protection (Myint *et al.*, 2019). More specifically, several emerging economies in Latin America, Asia and Africa are working towards expanding social health protection through the implementation of publicly funded health insurance (PFHI) schemes (Lagomarsino *et al.*, 2012a). PFHI is sometimes wholly subsidized by the government through general taxation or includes an element of premium contribution by beneficiaries. Emerging evidence clearly indicates that the expansion of PFHI results in concrete progress towards UHC, increasing access to care and enhancing financial protection through the removal of financial barriers at the point of care (Spaan *et al.*, 2012; Lagomarsino *et al.*, 2012b; Evans *et al*., 2013; Lu and Chiang, 2018; Erlangga *et al.*, 2019).

A critical factor for the success of any health scheme is the awareness of beneficiaries about the scheme and its services. Beneficiary awareness represents a crucial step towards ensuring that PFHI meets its promise of enhancing access to health services and financial protection for everyone. A deeper understanding of beneficiaries’ awareness and how it could be improved is essential for advancing progress towards UHC. Lack of knowledge about health programmes—especially those targeting vulnerable populations—impedes the right to access health, which is a basic human right (Sen, 2008). In this sense, a lack of awareness about one’s entitlements under programmes to advance UHC has been shown to affect the uptake and ultimately success of these programmes (Jacobs *et al.*, 2012; Platteau and Ontiveros, 2013; Panda *et al.*, 2016; Bocoum *et al.*, 2019). Evidence shows that the ultra-poor are often unable to take full advantage of free services offered at primary health facilities because of barriers including lack of access to information, low health awareness and exclusion from social and health institutions (BRAC, 2001; Ahmed *et al.*, 2006).

India has made substantial steps towards UHC with many Indian states launching their state-level PFHI and through the previously implemented national PFHI, ‘Rashtriya Swasthya Bima Yojana’ (RSBY, launched in 2008), followed by the ‘Pradhan Mantri Jan Arogya Yojana’ (PM-JAY) launched in 2018 (La Forgia and Nagpal, 2012). PM-JAY has been presented as an important step towards UHC, being the largest, fully government-funded scheme in the world enabling access to secondary and tertiary inpatient health-care services and associated financial protection to more than 500 million poor and vulnerable Indians (Angell *et al.*, 2019; Reddy *et al.*, 2020). Registration for PM-JAY is completely cashless (i.e. without any registration fee or prepayment) and, compared with most PFHI schemes, is not through annual enrolment drives tied to premium-based policy cycles. Instead, it is defined on the basis of households (HHs) fulfilling deprivation and occupational criteria identified by the government through the Socio-Economic Caste Census of 2011 (SECC 2011). Indian states can simultaneously use additional entitlement criteria to include other population groups, and former enrollees of RSBY are automatically included (Government of India, no date). Individuals are required to undergo a verification procedure to prove their identity and eligibility status before they can be formally registered as beneficiaries and avail benefits under the scheme. PM-JAY is currently running in 33 out of 36 states and Union Territories across India. Individuals can undergo the verification procedure at any time at designated government centres or at the time of presenting to a hospital with an illness. Hence, the awareness of the entitlement in the context of PM-JAY is crucial in order for the target population to utilize the scheme.

While the RSBY scheme included an enrolment process for the eligible population, under which an annual fixed prepayment of Indian Rupees (INR) 30 (about USD 0.4) was paid by beneficiaries during an enrolment camp or drive to be formally registered, PM-JAY simplified this step. The only way to benefit from the scheme is to be aware of one’s eligibility and related entitlements. However, evidence shows that in states with higher levels of poverty, beneficiaries are less likely to know about their PM-JAY entitlements (Smith *et al.*, 2019). At the moment, there is a growing body of evidence from India on the positive association between beneficiaries’ awareness of RSBY and subsequent enrolment in the scheme; yet, there were knowledge gaps about RSBY and its features even among the enrolled in remote areas and low community awareness about the main features and details of RSBY (Das and Leino, 2011; Nandi *et al.*, 2012; Rathi *et al.*, 2012; Moradhvaj and Saikia, 2019). On the other hand, there is scarce evidence about the role of awareness in accessing benefits under PM-JAY, given that the scheme is still relatively new. Reddy *et al.* (2020) found low awareness and readiness for implementing PM-JAY among health-care workers; health-care workers with higher awareness of PM-JAY had higher readiness to implement PM-JAY. Other researchers assessed beneficiaries’ awareness merely during the early stages of the scheme implementation using a very small sample of respondents; the results showed that awareness of the scheme among beneficiaries is still questionable, especially in urban areas (Kanore and Satpute, 2019).

This study contributes to filling this gap in knowledge by investigating beneficiaries’ awareness of the scheme and knowledge about the features and benefits offered by PM-JAY in six Indian states. The primary objective of the study is to shed light on individual and HH determinants of awareness of PM-JAY. The secondary objective is to explore the determinants of awareness of one’s eligibility for PM-JAY.

## Materials and methods

### Study setting

PM-JAY provides coverage up to INR 500 000 (about USD 6800) per family per year without restriction on family size, age or gender. PM-JAY is fully financed by the government for secondary and tertiary care hospitalization. In addition, up to 3 days of pre-hospitalization and 15 days post-hospitalization expenses, such as for diagnostics and medicines, are included. The Indian Government has educated eligible beneficiaries through Information, Education and Communication (IEC) activities initiated immediately after the scheme approval in 2018. A detailed communication strategy has been developed by the PM-JAY nodal organization, the National Health Authority (NHA), and implemented at both national and state levels (Government of India, 2018). The awareness campaigns not only included a variety of media such as billboards, radio and TV commercials but also targeted health education campaigns and community mobilization through Accredited Social Health Activists (ASHA) who are community outreach workers in the public health system and at the panchayat (local government) level (Bakshi *et al.*, 2018). One of the main IEC initiatives was a personalized letter sent from the Prime Minister (PM) to many eligible beneficiary families to make them aware of their entitlements under PM-JAY. In addition, an electronic record–based beneficiary identification system (BIS) has been implemented to allow anyone to check and consequently verify their eligibility status. In principle, individuals could confirm their eligibility in one of the several ways: in person at empanelled public or private hospitals, by telephone or online (through the ‘Am I eligible’ portal of the official PM-JAY webpage).

While beneficiaries can come to know about their eligibility status telephonically or through online means, they are required to physically verify their eligibility prior to availing services under the scheme. For physical eligibility verification, an operator searches for a potential beneficiary in the BIS using the beneficiaries’ name and address, the ration card number (ration cards are an official document issued by state governments in India to HHs that are eligible to purchase subsidized food grain from the Public Distribution System under the National Food Security Act), the mobile phone number or the PM’s letter (a letter from PM was sent to each eligible family through postal services). If the beneficiary’s name is found in the BIS, the operator sends the record to the insurance company, third-party administrator or state nodal agency for the final approval. After approval, a card is printed with a unique PM-JAY ID and given to the beneficiary as a proof of verification (even though a physical entitlement card is not mandatory to access services). The verification process is performed separately for each member of a HH and can be done at any moment without the need to be hospitalized or just before admission to the hospital (Government of India, no date). The need for physical verification prior to hospitalization makes awareness of one’s eligibility all the more crucial for accessing benefits under PM-JAY.

### Sampling and data collection strategies

For this study, we used data from a cross-sectional HH survey embedded within the framework of a larger evaluation of PM-JAY, conducted between the end of December 2019 and February 2020 in seven Indian States: Bihar, Chhattisgarh, Gujarat, Kerala, Meghalaya, Tamil Nadu and Uttar Pradesh. The start of the SARS-CoV-2 epidemic prevented completion of data collection in Kerala and thus Kerala is excluded from our study sample.

The detailed sampling strategy of the larger PM-JAY evaluation is available in the study by De Allegri *et al.* (2020). States and districts within states were purposively selected by policy stakeholders in consultation with study funders [the Deutsche Gesellschaft für Internationale Zusammmenarbeit (GIZ) GmbH] to reflect geographical diversity and the different models through which PM-JAY is being administered [through autonomous agencies (Bihar, Uttar Pradesh), insurance companies (Meghalaya) or a combination of the two (Chhattisgarh, Gujarat and Tamil Nadu)]. In each state, two or three districts with the best and average programme performance were selected, which are shown in Table 1.

**Table 1.** Sampled states and districts

State	Districts	Previously implemented PFHI scheme in 2018
Bihar	Muzaffarpur, Patna and Gaya	None
Chhattisgarh	Bilaspur and Raigarh	Mukhyamantri Swasthya Bima Yojana
Gujarat	Ahmedabad and Surat	Mukhyamantri Amrutam
Meghalaya	East Khasi Hills and South West Garo Hills	MHIS
Tamil Nadu	Coimbatore and Sivagangai	CMCHIS
Uttar Pradesh	Allahabad, Ghazipur and Rampur	None

At the district level, a district representative sample of eligible PM-JAY beneficiaries was randomly selected using the 2011 SECC data from 50 administrative blocks (the lowest administrative division in India), which served as primary sampling units (PSUs) within each district. We used age, gender and marital status to ensure district representativeness. Urban PSUs that had more than 7500 inhabitants were excluded because of the difficulty in identifying HHs with poor HH identification information, such as incomplete addresses and telephone numbers, as evidenced by the difficulty in tracing sampled urban HHs in the pilot study conducted in June 2019. Then, we randomly selected 15 HHs from each selected PSU to reach a total of 750 HHs per district. A total of 11 618 HHs were included in the final study sample.

A quantitative survey was administered by trained interviewers using Computer-Assisted Personal Interviewing software on digital devices. The survey was translated into all relevant local languages and administered by local interviewer teams fluent in these languages. Written informed consent was obtained from all respondents. For this specific analysis, only survey modules concerning socio-economic and demographic characteristics and awareness of PM-JAY were analysed.

### Variables and their measurement

#### Outcome variables

Table 2 shows our primary outcome variables. The first was a dichotomous variable aimed at measuring general awareness of the scheme. This variable was constructed on the basis of a single question asking an adult HH representative (usually the HH head) about their awareness of the PM-JAY scheme (‘Do you know about PM-JAY?’). The second primary outcome variable was a dichotomous variable constructed on the basis of a single question asking an adult HH representative about their awareness of their eligibility as an entire HH for PM-JAY (‘Is your family eligible?’). In this way, we wanted to compare different aspects of awareness of the scheme (general awareness of the scheme and awareness of one’s eligibility) along the explanatory variables.

**Table 2.** Variables: description, measurement and expected relationship with awareness on PM-JAY

Outcome variables	Measurement	%	*N*	Expected sign of association with the outcome variable
Knows of PM-JAY	0 = No	38.5	4471	NA
	1 = Yes	61.5	7147	
Knows family is eligible	0 = No, do not know	24.0	1561	NA
	1 = Yes	75.9	4922	
Explanatory variables: demographic variables				
Age: adult HH representative age	0 = <30 years 1 = 31–40	15.4 24.5	1794 2851	The relationship could be either positive or negative
	2 = 41–50	24.1	2797	
	3 = 51–60	17.8	2064	
	4 = > 61	18.2	2112	
Sex: adult HH representative sex	0 = Male 1 = Female	45.4 54.6	5270 6348	(+) male (−) female
Marital status: adult HH representative marital status	0 = Single 1 = Married	4.5 79.4	525 9227	(−) widowed/separated/ divorced
	2 = Widowed/separated/divorced	16.1	1866	
HH size	0 = <5 members 1 = Bigger families (5 or more members)	65.7 34.3	7633 3985	(+) bigger families
Explanatory variables: socio-economic variables				
Religion: adult HH representative religion	0 = Hindu 1 = Muslim 2 = Christian	83.9 5.4 9.8	9744 631 1139	There is no clear expected relationship between awareness and religion
	3 = Others (Sikhism, Jainism, Buddhism, Parsi/Zoroastrianism, others)	0.9	104	
Social group: adult HH representative caste	0 = Others/general castes or no caste 1 = Scheduled Caste	6.6 33.8	762 3927	(±) Marginalized caste groups could be more aware of the scheme as main target of PM-JAY
	2 = Scheduled Tribe	27.0	3131	
	3 = Other Backward Class	32.7	3798	
Education: adult HH representative education level	0 = Illiterate 1 = Completed primary	56.9 20.7	5727 2083	(−) Illiterate (+) above secondary
	2 = Completed secondary	18.1	1824	
	3 = Above secondary	4.2	424	
Occupation: adult HH representative employment status	0 = Not employed 1 = Self-employed	41.1 20.1	4777 2340	(−) self-employed, daily wage worker (+) regular salaried job
	2 = Daily wage worker	29.2	3395	
	3 = Regular salaried job	5.9	690	
	4 = Others	3.6	416	
Monthly HH consumption expenditure (Rs)	0 = First quartile (poorest) 1 = Second quartile 2 = Third quartile 3 = Fourth quartile (wealthiest)	21.0 23.3 25.2 30.6	2435 2705 2924 3554	(−) first quartile of consumption expenditure (+) fourth quartile of consumption expenditure

Table 3 shows our secondary outcomes related to eligible individuals’ knowledge of specific scheme features, as well as sources and channels of information asked to respondents with positive general scheme awareness. We did not ask further details about the features of the scheme to HHs in Tamil Nadu since PM-JAY and the existing state scheme, Chief Minister’s Comprehensive Health Insurance Scheme (CMCHIS), were merged and the scheme now operates as the co-branded PM-JAY-CMCHIS. Consequently, all beneficiaries of CMCHIS were included in this new scheme and IEC activities in Tamil Nadu state pertained to specific features of this co-branded scheme, not specifically to PM-JAY.

**Table 3.** Awareness of PM-JAY

Channels of getting to know about PM-JAY^a^ (multiple answers were allowed)	%	*N*
Friends	59.0	7147
ASHA workers	54.2	7147
PM’s letter	29.9	7147
Panchayat	12.0	7147
Anganwadi workers	11.0	7147
TV	7.6	7147
Hospitals	7.1	7147
Ayushman Bharat Diwas^c^	4.5	7147
Kiosk	1.8	7147
Radio	1.3	7147
Health camps	1.3	7147
Arogya Mitra	1.1	7147
Others^d^	6.9	7147
**Features of PM-JAY** ^b^ **(multiple answers were allowed)**		
Hospitalization	55.0	6483
Coverage of Rs 500 000 per family per year	31.7	6483
Includes pre- and post-hospitalization expenses	5.8	6483
No cap on family size	3.8	6483
Transportation allowance not included	2.0	6483
Portability of benefits across states	0.7	6483
Do not know	31.8	6483

a The question is asked to all adult HH representatives who replied ‘yes’ to the question about awareness of PM-JAY.

b The question is asked to all adult HH representatives who replied ‘yes’ to the question about family eligibility.

c Ayushman Bharat Diwas is an event celebrated across India on 30 April to promote the benefits of the scheme to the eligible population.

d Others include the following categories: website, call centre, leaflets/brochures, camps organized in village/slum, insurance providers, auxiliary nurse midwife, community educators, NGO, SMS, hoarding/billboards and any other.

#### Explanatory variables

We included variables capturing demographic, socio-economic and geographic location that can influence access to information and awareness: adult HH representatives’ sex, age, marital status, HH size (demographic); religion, caste, educational attainment and employment status, HH monthly expenditure on consumption (socio-economic) and geographical location (states). Caste groups were categorized based on the respondent’s self-reported affiliation to one of the groups used by the Government of India: other/general castes or no caste, Scheduled Caste, Scheduled Tribe and Other Backward Classes (National Commission for Backward Classes and Government of India, 1980). For educational attainment, we distinguished between adult HH representatives who are illiterate, completed primary school (from the 1st to the 7th grade), secondary school (up to grade 10) and higher than secondary school. Employment status was categorized as adult HH representatives primarily employed in regular salaried jobs, self-employed (both in the agricultural and non-agricultural sectors), daily wage workers (both in the agricultural and non-agricultural sectors), not currently employed and other sectors. We used HH’s monthly consumption expenditure expressed in INR as a proxy for income to measure HH wealth; we estimated quartiles of HH consumption expenditure on the basis of the total state-specific consumption expenditure. The HH consumption expenditure was computed at the HH level by adding the food expenditure incurred in the last 30 days and the non-food expenditure of the last 365 days divided by 12 to obtain the average monthly expenditure.

### Analytical approach

First, we used descriptive statistics to illustrate the distribution of the full set of outcome variables and to identify how many individuals were aware of PM-JAY. Second, we used bivariate analysis and chi-squared (${\chi ^2}$) tests to investigate whether the observed differences between the two groups (respondents aware of PM-JAY vs. the unaware) were statistically significant. We estimated two sets of logistic regressions to confirm any statistical association between PM-JAY awareness and awareness about the eligibility for PM-JAY and the respective socio-economic (religion, caste, education, occupation and wealth) and demographic (age, sex, marital status and HH size) explanatory variables, as well as state of residence. The regressions were estimated with robust standard errors clustered at the district level (14 districts in total). As explanatory variables, we included all demographic and socio-economic variables that displayed a statistically significant difference in the chi-squared test between the two groups of HHs. The analysis was conducted using Stata 15.


$$\eqalign{\Pr \left( {y = 1} \right) & = F\left( {{\beta _0} + {\beta _1}{\rm{demographic}} + {\beta _2}{\rm{socio - economic}}}\right. \\ & \quad + \left.{{\beta _3}{\rm{geographical}}} \right)}$$

where $F\left( z \right)\, = \,{e^z}/\left( {1\, + \,{e^z}} \right)$ is the cumulative logistic distribution and *y* is the PM-JAY awareness; awareness about PM-JAY eligibility.

## Results

### Descriptive results

Table 2 describes the outcome and explanatory variables. Out of 11 618 adult HH representatives, 62% reported being aware of PM-JAY (*N* = 7147). Out of the 7147 aware HH representatives, 6483 were further asked if they knew about their PM-JAY eligibility status, which was confirmed by 76% (*N* = 4922). As explained in the Methods section, we did not collect information about awareness of one’s eligibility status in Tamil Nadu; hence, 664 aware HHs from Tamil Nadu were excluded.

The 7147 HH representatives who were aware of PM-JAY were further asked about the main source of learning about PM-JAY. The most frequently mentioned sources included friends and family (59%), ASHA workers (54.2%) and the PM’s letter (29.9%) (see Table 3).

The 6483 HH representatives aware of their eligibility status were further asked about scheme features. On average, the most well-known features of the scheme were that PM-JAY covered hospitalization expenses (55.0%), with a coverage amount of Rs 500 000 per family per year (31.7%), and that pre- or post- hospitalization expenses were included (5.8%). However, 31.8% of respondents could not recall any feature of the scheme (see Table 3).

### Bivariate analysis

The chi-squared tests in Table 4 show significant differences in the distribution of the outcome variable ‘awareness of PM-JAY’ across all explanatory variables. A higher proportion of female respondents were found in the unaware group than in the aware group. Most respondents were married (80.8% aware of PM-JAY and 77.2% not aware of PM-JAY), with a slightly higher share of widowed/separated or divorced respondents in the unaware group (18.7%) compared to the aware group (14.4%). Most adult HH representatives aware of the scheme belonged to Other Backward Class (37.1%) followed by a Scheduled Caste (36.7%), while respondents unaware of the scheme were more frequently from a Scheduled Tribe (38.0%) followed by from a Scheduled Caste (29.1%). A higher proportion of unaware of PM-JAY HH representatives were illiterate compared to those who were aware of the scheme (54.8% in the aware group; 60.6% in the unaware group). Larger shares of monthly HH expenditure falling in the poorer quartiles were found in respondents unaware of the scheme even though the largest share in both the aware and unaware groups was in the richest quartile. Among the aware group, there were a higher proportion of HH representatives from districts in Gujarat and Uttar Pradesh, while among the unaware group, a higher proportion of respondents were from districts in Meghalaya and Tamil Nadu.

**Table 4.** Bivariate analysis and chi-squared results for awareness categories

	Aware of PM-JAY		Not aware of PM-JAY		Total			
	%	*N*	%	*N*	%	*N*	Chi-square value	*P*-value
**Demographic variables**								
Age, years							40.460	0.000
30 or less	15.6	1114	15.2	680	15.4	1794		
31–40	25.2	1803	23.4	1048	24.5	2851		
41–50	25.1	1797	22.4	1000	24.1	2797		
51–60	17.5	1250	18.2	814	17.8	2064		
More than 61	16.6	1183	20.8	929	18.2	2112		
Total	100	7147	100	4471	100	11 618		
Sex							40.949	0.000
Male	47.7	3409	41.6	1861	45.4	5270		
Female	52.3	3738	58.4	2610	54.6	6348		
Total	100	7147	100	4471	100	11 618		
Marital status							37.410	0.000
Single	4.8	341	4.1	184	4.5	525		
Married	80.8	5774	77.2	3453	19.4	9227		
Widowed/separated/divorced	14.4	1032	18.7	834	16.1	1866		
Total	100	7147	100	4471	100	11 618		
HH size							33.253	0.000
<5	63.7	4552	68.9	3081	65.7	7633		
5 or more	36.3	2595	31.1	1390	34.3	3985		
Total	100	7147	100	4471	100	11 618		
**Socio-economic variables**								
Religion							0.000	0.000
Hindu	90.7	6485	72.9	3259	83.8	9744		
Muslim	6.2	442	4.2	189	5.4	631		
Christian	2.9	205	20.9	934	9.8	1139		
Others	0.2	15	2.0	89	0.9	104		
Total	100	7147	100	4471	100	11 618		
Social group							491.181	0.000
Scheduled Caste	36.7	2626	29.1	1301	33.8	3927		
Scheduled Tribe	20.0	1431	38.0	1700	27.0	3131		
Other Backward Class	37.1	2649	25.7	1149	32.7	3798		
Others/general castes, no caste	6.2	441	7.2	321	6.6	762		
Total	100	7147	100	4471	100	11 618		
Education							91.401	0.000
Illiterate	54.8	3463	60.6	2264	56.9	5727		
Primary	20.5	1293	21.2	790	20.7	2083		
Secondary	19.3	1221	16.1	603	18.1	1824		
Above secondary	5.5	346	2.1	78	4.2	424		
Total	100	6323	100	3735	100	10 058		
Occupation							15.989	0.000
Not employed	41.3	2951	40.8	1826	41.1	4777		
Self-employed	20.7	1481	19.2	859	20.1	2340		
Daily wage worker	28.7	2051	30.1	1344	29.2	3395		
Regular salaried job	6.1	439	5.6	251	5.9	690		
Others	3.2	225	4.3	191	3.6	416		
Total	100	7147	100	4471	100	11 618		
Monthly HH consumption expenditure (INR)							39.629	0.000
First quartile (poorest)	19.4	1386	23.5	1049	21.0	2435		
Second quartile	22.7	1624	24.2	1081	23.3	2705		
Third quartile	26.9	1856	23.9	1068	25.2	2924		
Fourth quartile (wealthiest)	31.9	2281	28.5	1273	30.6	3554		
Total	100	7147	100	4471	100	11 618		
**HH location**							0.000	0.000
Bihar	21.7	1553	23.2	1036	22.3	2589		
Chhattisgarh	12.0	855	14.2	633	12.8	1488		
Gujarat	22.4	1600	2.4	109	14.7	1709		
Meghalaya	3.5	252	28.0	1251	12.9	1503		
Tamil Nadu	9.3	664	24.2	1082	15.0	1746		
Uttar Pradesh	31.1	2223	8.1	360	22.2	2583		

### Logistic regression results of determinants of awareness and eligibility

Table 5 shows both unadjusted (bivariate) and adjusted (multivariate) odds ratio (OR) estimates of the association between awareness of PM-JAY as well as its eligibility and demographic and socio-economic characteristics of the adult HH representatives. The unadjusted results in Column 1 do not differ from the statistical association results reported in Table 4. The coefficients from the adjusted logit regression in Column 2 for age of the respondent, education, salaried occupation and residing in districts of Gujarat or Uttar Pradesh were statistically significant at the 5% or 1% level and had positive signs. The adjusted OR for respondents in the age group of 41–50 years was 1.30 (*P*-value < .05), and in the age group of 51–60 years, it was 1.29 (*P*-value < 0.01), compared to the age group of under 30 years. The adjusted OR for awareness increased from 1.26 *P*-value < 0.01) for those with primary education to 2.52 (*P*-value < 0.01) for those with higher than secondary education, compared to illiterate respondents. These results imply that an increase in the age and educational attainment of the respondents impacted positively the odds of being aware of PM-JAY compared to their reference category (age ≤ 30 years and being illiterate, respectively), adjusting for all other covariates. The adjusted OR for salaried respondents was 1.60 when compared to unemployed respondents (*P*-value < 0.05). Women respondents were less likely to be aware of PM-JAY (adjusted OR 0.83, *P*-value < 0.1) than men. Furthermore, respondents who resided in districts of Gujarat and Uttar Pradesh were 13.88 (*P*-value < 0.01) and 4.72 (*P*-value < 0.01) times, respectively, more likely to know about the scheme compared to respondents from districts in Bihar. Residing in districts of Meghalaya and Tamil Nadu affected negatively the odds of knowing about PM-JAY. On average, respondents from districts in these states were 0.23 (*P*-value < 0.01) and 0.30 (*P*-value < 0.05) times less likely to be aware of the scheme compared to respondents from districts of Bihar, respectively.

**Table 5.** Logit regression analysis on awareness of PM-JAY and awareness of eligibility for PM-JAY

	Do you know about PM-JAY (yes = 1, no = 0)						Is your family eligible (yes = 1, no/do not know = 0)					
	Unadjusted (1)			Adjusted (2)			Unadjusted (3)			Adjusted (4)		
	OR	95% CI		OR	95% CI		OR	95% CI		OR	95% CI	
**Age, years**												
≤30	1			1			1			1		
31–40	1.72^*^	0.97	3.04	1.19^*^	1.00	1.42	3.03^***^	1.84	4.98	1.05	0.82	1.33
41–50	1.80^*^	0.93	3.46	1.30**	1.02	1.66	3.12^***^	1.94	5.02	0.98	0.75	1.29
51–60	1.54	0.78	3.04	1.29^***^	1.08	1.54	3.62^***^	2.31	5.67	1.24	0.92	1.68
≥61	1.27	0.72	2.27	1.22	0.96	1.57	3.39^***^	1.98	5.83	1.35^*^	0.96	1.90
**Sex**												
Male	1			1			1			1		
Female	1.43	0.76	2.69	0.83^*^	0.67	1.03	3.50^***^	2.29	5.34	1.29	0.92	1.81
**Marital status**												
Single	1			1			1			1		
Married	1.67^*^	0.94	2.97	0.87	0.61	1.23	3.15^***^	1.97	5.04	0.93	0.66	1.30
Widow/separated/divorced	1.24	0.62	2.48	0.78^*^	0.59	1.04	3.44^***^	2.21	5.35	0.89	0.56	1.42
**HH size**												
<5 members	1			1			1			1		
5 or more	1.87^*^	0.96	3.63	0.87^*^	0.74	1.02	3.36^***^	2.13	5.31	0.84^*^	0.70	1.01
**Religion**												
Hindu	1			1			1			1		
Muslim	2.34^***^	1.33	4.13	0.62^***^	0.53	0.74	4.40^***^	1.93	10.00	1.06	0.67	1.67
Christian	0.22^***^	0.18	0.26	0.96	0.66	1.39	2.78^***^	1.56	4.96	0.79	0.46	1.35
Others	0.17^***^	0.14	0.20	1.05	0.50	2.21	1.50^*^	0.93	2.43	1.83^***^	1.19	2.83
**Social group**												
Others/general castes, no caste	1			1			1			1		
Scheduled Caste	2.02**	1.16	3.52	1.28	0.88	1.86	3.10^***^	1.91	5.05	1.25	0.88	1.78
Scheduled Tribe	0.84	0.26	2.77	0.62	0.31	1.24	2.78^***^	1.44	5.38	0.93	0.65	1.32
Other Backward Class	2.31**	1.20	4.43	1.21	0.86	1.68	3.57^***^	1.96	6.52	1.29**	1.01	1.66
**Education**												
Illiterate	1			1			1			1		
Primary	1.64	0.72	3.71	1.26^***^	1.12	1.43	3.18^***^	1.73	5.86	1.06	0.94	1.20
Secondary	2.02**	1.01	4.07	1.59^***^	1.27	1.97	3.88^***^	1.61	5.17	1.04	0.83	1.31
Above secondary (12+)	4.44^***^	1.97	10.00	2.52^***^	1.78	3.57	3.23^***^	1.54	6.81	1.08	0.73	1.59
**Occupation**												
Not employed	1			1			1			1		
Self-employed	1.72*	0.91	3.26	1.05	0.87	1.26	2.67^***^	1.46	4.87	1.14	0.70	1.83
Daily wage worker	1.53	0.74	3.15	1.10	0.82	1.47	3.49^***^	2.15	5.66	1.21	0.91	1.60
Regular salaried Job	1.75	0.87	3.50	1.60**	1.07	2.39	3.25^***^	1.76	6.02	1.04	0.63	1.73
Others	1.18	0.34	4.04	1.05	0.62	1.79	2.95**	1.21	7.17	0.73	0.31	1.74
**Monthly HH** **consumption expenditure (Rs)**												
First quartile (poorest)	1			1			1			1		
Second quartile	1.50	0.80	2.84	1.13	0.93	1.37	3.42^***^	1.92	6.11	1.57^***^	1.14	2.15
Third quartile	1.74*	0.99	3.06	1.32*	0.96	1.82	3.61^***^	2.22	5.85	2.01^***^	1.48	2.74
Fourth quartile (wealthiest)	1.79	0.87	3.70	1.47	0.92	2.35	3.65^***^	2.27	5.89	2.25^***^	1.37	3.68
**HH geographical location**												
Bihar	1			1			1			1		
Chhattisgarh	1.35	0.91	2.00	0.97	0.68	1.39	1.01	0.75	1.36	0.42^***^	0.28	0.63
Gujarat	14.68^***^	10.69	20.16	13.88^***^	9.24	20.84	6.34^***^	4.59	8.76	3.22^***^	2.28	4.54
Meghalaya	0.20^***^	0.18	0.22	0.23^***^	0.12	0.43	2.94^***^	1.91	4.52	2.48^***^	1.42	4.34
Tamil Nadu	0.61	0.28	1.36	0.30^***^	0.15	0.62	NA	NA	NA	NA	NA	NA
Uttar Pradesh	6.18^***^	3.75	10.16	4.72^***^	3.04	7.34	3.42^***^	1.94	11.58	2.29	0.83	6.34
*N*				10 058						5831		

^a^ Robust standard errors clustered at the district level (14 districts).

*** *P* < 0.01; **P < 0.05; *P < 0.10.

CI = confidence interval.

Respondents in Tamil Nadu were not asked questions about eligibility, verification and knowledge of the features of PM-JAY.

Due to some missing values, the number of observations is smaller than the total sample number.

We report, in Column 3, the results of the unadjusted logistic regressions using ‘is your family eligible’ as the dependent variable. The coefficients from the adjusted logit regression displayed in Column 4 show that respondents ascribing to other religious faiths, part of Other Backward Classes, with higher levels of wealth and residing in districts of Gujarat and Meghalaya were statistically significant at 5% or 1% and had positive signs. An increase in wealth caused an increase on the odds of being aware of their own eligibility status compared to those in the first quartile of wealth, with adjusted ORs of knowing about eligibility increasing from 1.57 (*P*-value < 0.01) in the second consumption quartile to 2.25 (*P*-value < 0.01) in the wealthiest consumption quartile, compared to the poorest quartile. Respondents from Other Backward Classes were 1.29 (*P*-value < 0.05) times more likely to know about their eligibility compared to respondents from a general caste or without a caste. Respondents from districts in Gujarat and Meghalaya were, respectively, 3.22 (*P*-value < 0.001) and 2.48 (*P*-value < 0.001) times more likely to be aware of their eligibility compared to respondents from districts in Bihar. On the other hand, respondents from districts in Chhattisgarh were 0.42 times (*P*-value < 0.01) less likely to know about their eligibility status compared to respondents from districts in Bihar, adjusting for all other covariates.

## Discussion

This study makes an important contribution to the existing literature as one of the very first studies addressing determinants of beneficiaries’ awareness of PM-JAY and of their eligibility status in India. Previous studies on RSBY have focused on examining determinants of enrolment in RSBY, while studies on PM-JAY have focused on preliminary findings on beneficiaries’ and medical staff awareness of the scheme (Das and Leino, 2011; Nandi *et al.*, 2012; Rathi *et al.*, 2012; Kanore and Satpute, 2019; Reddy *et al.*, 2020). Awareness about health insurance is critical for beneficiary participation and impacts enrolment, utilization of health services, improvements in health status and reduction in health expenditures (Acharya *et al.*, 2012), improved productivity and welfare effects (Barnes *et al.*, 2017), in addition to spillover effects like peer-to-peer information dissemination affecting all the prior effects (Chatterjee *et al.*, 2018). To the authors’ knowledge, no explicit, large-scale examination of determinants of scheme and eligibility awareness of PM-JAY in India has been reported so far.

Our findings reveal that 14–18 months after implementation, three out of five adult HH representatives knew about the scheme, which is quite positive in the early stages of a scheme; however, there is still considerable scope to increase general awareness of PM-JAY. Furthermore, among respondents who were aware of PM-JAY, less than four out of five knew about their eligibility for the scheme. The results indicate that the channels of communication employed to disseminate information about PM-JAY are not reaching all intended beneficiaries. However, when beneficiaries are aware about the scheme—through family and friends, ASHA workers and the PM’s letter—the awareness about their own eligibility for PM-JAY is on average high. Nonetheless, respondents knowledge of the features suggests that the information delivered to them is incomplete or unclear. A third of respondents who were aware of PM-JAY and of their own eligibility in the scheme did not know what kind of benefits they were entitled to: in other words, they knew about their scheme eligibility, but in essence, they lacked the knowledge of what particular benefits PM-JAY had to offer them. This calls for more effective awareness campaigns to reach all beneficiaries—especially the most vulnerable—and to deliver complete and clear knowledge on the benefits offered. In light of the literature about beneficiaries’ awareness and UHC, these results suggest a possible detrimental effect of lack of awareness on the scheme utilization and consequently on the success of the scheme (Giedion *et al.*, 2013).

We observe socio-economic gradients in respondents awareness of PM-JAY as well as awareness of their eligibility. Even though the association between awareness and gender was not highly significant, women were still less likely to be aware of PM-JAY than men. This is substantiated by the growing evidence on women’s exclusion from financial protection mechanisms for health in India (Jain, 2012; Nandi *et al.*, 2013; 2017; Kansra, 2015; Karpagam *et al.*, 2016; Shaikh *et al.*, 2018; Moradhvaj and Saikia, 2019; RamPrakash and Lingam, 2021). PM-JAY design sought to do away with HH member limits for enrolment, which was found to adversely affect the enrolment of women HH members, and our findings suggest that this merits policy attention to ensure that women are aware of the scheme and subsequently their entitlements (Karpagam *et al.*, 2016). Low awareness of women is likely to facilitate their exclusion from enrolment in health insurance, which should be verified by further research.

We find that Other Backward Classes are much more likely to know about their eligibility status than those belonging to general or no castes. An earlier study on district-level enrolment in RSBY found that districts with a higher share of Other Backward Classes were less likely to participate in RSBY and had lower enrolment rates, and therefore, higher awareness of one’s eligibility in this group in our study is a positive sign (Nandi *et al.*, 2013).

Increasing educational attainment was positively associated with being aware of PM-JAY, but not with knowledge of eligibility status. On the other hand, increasing HH wealth was not associated with general awareness about the scheme but exhibited significant gradients with knowledge of eligibility. Other studies also support education and wealth gradients in health insurance scheme awareness and awareness of eligibility (Kirigia *et al.*, 2005; De Allegri *et al.*, 2006; Bourne and Kerr-Campbell, 2010).

The most significant driver of differences in knowledge of PM-JAY and of one’s eligibility was the state of residence. This is not surprising, given that Indian states are socially, geographically, linguistically and culturally diverse and also have varied histories with the implementation of different statutory health insurance schemes. Furthermore, while PM-JAY guidelines prescribe a reference of IEC activities for beneficiaries, it is up to the states to implement them. We observe that respondents from Meghalaya and Tamil Nadu, states that had statutory health insurance schemes implemented prior to or in close conjunction with PM-JAY, had lower awareness of PM-JAY than in Bihar. This may be partially due to confusion about the different names of PM-JAY in the states, although we included the alternative nomenclatures in translated survey questions. We chose Bihar as a baseline comparator because while the RSBY had been implemented in the state, it was stopped in 2015, providing a period of 3 years with blank canvas of no implementation of any scheme before PM-JAY was launched. Likewise, Uttar Pradesh also had no large statutory health insurance scheme after RSBY was stopped there in 2015. However, we observe that respondents from Uttar Pradesh were almost five times more likely to be aware of PM-JAY than those from Bihar, suggesting differences in dissemination efforts in these states with similar starting conditions. This may be also a reflection of the capacity of the state health agencies in these states, which are responsible for implementing PM-JAY in the state. Respondents from Gujarat were almost 15 times more likely to know about PM-JAY than those from Bihar; this may possibly be due to the proximity of state and national political leadership and subsequent prioritization of awareness efforts in the state. Furthermore, although Gujarat had earlier implemented a statutory health insurance scheme, the Ma Amrutam Yojana, this scheme was fully integrated into the implementation of the new PM-JAY. We hypothesize that more cohesive dissemination efforts for PM-JAY may have overcome potential confusion between the two schemes in potential beneficiaries. In contrast, respondents in states like Meghalaya and Tamil Nadu with pre-existing state schemes, the Megha Health Insurance Scheme (MHIS) and the CMCHIS, respectively, exhibit much lower awareness than those in Bihar. The MHIS was first implemented for socially and economically vulnerable sections of society and subsequently expanded to provide universal coverage to all Meghalaya citizens. With the implementation of PM-JAY, state citizens who did not meet PM-JAY eligibility criteria continued to be enrolled in the MHIS without needing to undergo PM-JAY verification processes. This may have obfuscated the need among all citizens to first, be aware of PM-JAY distinct from MHIS, and second, to know about PM-JAY entitlements and verification processes. Overall dissemination efforts in Meghalaya could be more challenging than in other states due to the geography of the state itself with villages spread over larger distances in mountainous terrain. However, we confirmed that even if respondents from Meghalaya were in the first place less likely to know of PM-JAY, the ones who were aware of the scheme were more likely to know about their eligibility status than respondents from Bihar. This may indicate that dissemination efforts regarding PM-JAY eligibility information have been more effective than in other states. In Tamil Nadu, as mentioned earlier, the popularity of the state scheme CMCHIS may have had detrimental effects on the awareness of the new PM-JAY. Furthermore, PM-JAY was introduced in Tamil Nadu by renaming the CMCHIS to PM-JAY-CMCHIS, and procedurally, the scheme continued to operate as per CMCHIS guidelines, obviating the need to disseminate information on changes affecting beneficiaries since there were none. This may explain the low awareness we see in respondents in Tamil Nadu. In Chhattisgarh, we observe that respondents are almost half likely to know about eligibility than respondents in Bihar, again possibly due to the co-existence of the Mukhyamantri Swasthya Bima Yojana, which is a universal statutory health insurance scheme providing basic coverage to all citizens of the state. Since all citizens are automatically covered by the state for some benefits, it reduces the need for HHs to be specifically aware of their PM-JAY eligibility.

### Methodological considerations

Albeit being the first large-scale study conducted to assess awareness of PM-JAY, this study suffers from a few limitations. First, our study does not include an urban sample. The sampling strategy of the larger evaluation study excluded beneficiaries from urban settings with more than 7500 inhabitants due to difficulties in tracking and identifying beneficiaries in such large settings with poor information on their addresses. The evaluation study is representative for PM-JAY beneficiaries from rural and small urban areas only. In the analysis of this paper, we excluded HHs sampled from the urban areas; therefore, the data and the results are representative only in regard to rural settings in the selected six states. Furthermore, we did not apply weights in our analysis. Since our sampling frame was the SECC 2011 database used for determining basic PM-JAY eligibility across states, using sampling weights would have ensured that our sample was representative of eligible HHs within sampled districts in 2011. Since our study was conducted between December 2019 and February 2020, we chose not to apply sampling weights. This also draws attention to the need for updating the underlying eligibility database for PM-JAY.

The second limitation concerns the awareness section. Information on PM-JAY, eligibility, source of knowledge and knowledge of the features was asked from only one adult respondent from each HH, usually the HH head. In this way, we assumed that the awareness of the other members of the family aligned with the main respondent. This implies that probably the most aware or autonomous member of the HH responded on behalf of the rest of the family; therefore, the awareness could be lower in other HH members.

Finally, it must be considered that quantitative studies can fail to measure and account for all the relevant variables and factors playing a role in affecting awareness. Further qualitative inquiry is needed to fully comprehend why one out of three eligible individuals remains unaware of the scheme and hence of one’s own eligibility.

This study provides the first large-scale evidence on individual and HH determinants of general awareness of PM-JAY and of one’s eligibility. We found that three out of five respondents knew about PM-JAY, and among those who knew, four out of five knew about their eligibility. However, these numbers show considerable scope for further outreach. Furthermore, eligible beneficiaries appeared to have inadequate information on specific scheme features, suggesting that they can miss out on maximizing their benefits under PM-JAY. While existing channels of communication such as family and friends, ASHAs and the PM’s letter had high recall value, more tailored approaches are needed focusing not only on general awareness but also on knowledge of scheme features that can empower beneficiaries to demand entitled services. We find evidence of sex-, caste- and educational attainment-related gradients in general awareness of PM-JAY and awareness of eligibility. State-specific differences in general awareness were also observed, with states with previously implemented PFHI having both lower (Tamil Nadu and Meghalaya) and higher (Gujarat) awareness than Bihar, which has had no functional PFHI since 2015. These state-specific differences suggest that the legacies of past schemes may be affecting the current awareness and knowledge of eligible beneficiaries and that tailored, state-specific IEC approaches are needed. Addressing these issues may help PM-JAY realize its full potential of achieving UHC for the marginalized Indian population.

## Data Availability

The data that support the findings of this study are available from Deutsche Gesellschaft für Internationale Zusammenarbeit GmbH but restrictions apply to the availability of these data, which were used under license for the current study, and so are not publicly available. Data are however available from the authors upon reasonable request and with permission of Deutsche Gesellschaft für Internationale Zusammenarbeit GmbH.

## Abbreviations

ASHA = Accredited Social Health Activists

BIS = beneficiary identification system

CMCHIS = Chief Minister’s Comprehensive Health Insurance Scheme

GIZ = Deutsche Gesellschaft für Internationale Zusammmenarbeit

IEC = Information, Education and Communication

INR = Indian Rupees

MHIS = Megha Health Insurance Scheme

NHA = National Health Authority

OR = odds ratio

PFHI = publicly funded health insurance

PM-JAY = Pradhan Mantri Jan Arogya Yojana

PSU = primary sampling unit

RSBY = Rashtriya Swasthya Bima Yojana

SDG = Sustainable Development Goal

SECC = Socio-Economic Caste Census

UHC = Universal Health Coverage

## Reflexivity statement

The analysis presented in this manuscript is the product of a collaboration between 11 authors, including people of different gender, nationality and seniority. Additionally, this manuscript is the product of a collaboration between people working in the academic and the policy sector. The most senior authors were responsible for drafting the initial study design, on which this specific analysis rests, but all authors, irrespective of gender, nationality and seniority, were actively engaged in the definition of the data collection tools, the strategy selected for this specific analysis, the interpretation of the findings and the writing of the manuscript. In fact, the two first co-authors are early career female researchers of two different nationalities. Active collaboration across all study phases was facilitated by the fact that the team members have known each other over a long period of time and have worked together, in different constellations, before.

## References

[R1] Acharya A, Vellakkal S, Taylor F et al. 2012. Impact of national health insurance for the poor and the informal sector in low- and middle-income countries. *Evidence for Policy and Practice Information and Co-ordinating Centre (EPPI-Centre)*. London.

[R2] Ahmed SM, Petzold M, Kabir ZN et al. 2006. Targeted intervention for the ultra poor in rural Bangladesh: does it make any difference in their health-seeking behaviour? *Social Science & Medicine* 63: 2899–911. 16954049 10.1016/j.socscimed.2006.07.024

[R3] Angell BJ, Prinja S, Gupt A et al. 2019. The Ayushman Bharat Pradhan Mantri Jan Arogya Yojana and the path to universal health coverage in India: overcoming the challenges of stewardship and governance. *PLoS Medicine* 16: e1002759. 10.1371/journal.pmed.1002759 PMC6405049 30845199

[R4] Bakshi H, Sharma R, Kumar P. 2018. Ayushman Bharat Initiative (2018): what we stand to gain or lose! *Indian Journal of Community Medicine* 43: 63. 10.4103/ijcm.IJCM_96_18 PMC5974836 29899601

[R5] Barnes K, Mukherji A, Mullen P et al. 2017. Financial risk protection from social health insurance. *Journal of Health Economics* 55: 14–29. 28619488 10.1016/j.jhealeco.2017.06.002

[R6] Bocoum F, Grimm M, Hartwig R et al. 2019. Can information increase the understanding and uptake of insurance? Lessons from a randomized experiment in rural Burkina Faso. *Social Science & Medicine* 220: 102–11. 30415141 10.1016/j.socscimed.2018.10.029

[R7] Bourne PA, Kerr-Campbell MD. 2010. Determinants of self-rated private health insurance coverage in Jamaica. *Health* 2: 541–50.

[R8] BRAC . 2001. Challenging the Frontiers of Poverty Reduction (CFPR) – targeting the ultrapoor – targeting social constraints, poverty programme proposal: Volume 1 & 2, March 2001. © BRAC.

[R9] BRAC 2001 Challenging the Frontiers of Poverty Reduction (CFPR) - targeting the ultrapoor- targeting social constraints, poverty programme proposal. Vol. 1 & 2. Dhaka, Bangladesh: © BRAC.

[R10] Chatterjee C, Joshi R, Sood N et al. 2018. Government health insurance and spatial peer effects: new evidence from India. *Social Science & Medicine* 196: 131–41. 29175702 10.1016/j.socscimed.2017.11.021

[R11] Das J, Leino J. 2011. Evaluating the RSBY: lessons from an experimental information campaign. *Economic and Political Weekly* 32: 85–93.

[R12] De Allegri M, Sanon M, Sauerborn R. 2006. “To enrol or not to enrol?”: a qualitative investigation of demand for health insurance in rural West Africa. *Social Science & Medicine* 62: 1520–7. 16169645 10.1016/j.socscimed.2005.07.036

[R13] De Allegri M, Srivastava S, Strupat C et al. 2020. Mixed and multi-methods protocol to evaluate implementation processes and early effects of the Pradhan Mantri Jan Arogya Yojana scheme in seven Indian states. *International Journal of Environmental Research and Public Health* 17: 1–15. 10.3390/ijerph17217812 PMC7663328 33114480

[R14] Erlangga D, Suhrcke M, Ali S et al. 2019. The impact of public health insurance on health care utilisation, financial protection and health status in low- and middle-income countries: a systematic review. *PLoS One* 14: e0219731. 10.1371/journal.pone.0219731 PMC6713352 31461458

[R15] Evans DB, Hsu J, Boerma T. 2013. Universal health coverage and health outcomes. *Bulletin of the World Health Organization.* 91: 546–546A. 23940398 10.2471/BLT.13.125450 PMC3738317

[R16] Giedion U, Alfonso EA, Díaz Y. 2013. The impact of universal coverage schemes in the developing world: a review of the existing evidence. Washington DC: World Bank.

[R17] Government of India . 2018. *Information, Education and Communication (IEC) Guidebook for SHAs*. New Delhi: National Health Authority.

[R18] Government of India . Policy Guidelines Ayushman Bharat. https://www.pmjay.gov.in/policy-and-guidelines; accessed 16 September 2018.

[R19] Jacobs B, Ir P, Bigdeli M et al. 2012. Addressing access barriers to health services: an analytical framework for selecting appropriate interventions in low-income Asian countries. *Health Policy and Planning* 27: 288–300. 21565939 10.1093/heapol/czr038

[R20] Jain K . 2012. Health Insurance in India: The Rashtriya Swasthya Bima Yojana Assessing Access for Informal Workers. Women in Informal Employment: Globalizing and Organizing (WIEGO) Policy Brief No. 10. Manchester. United Kingdom. https://www.wiego.org/sites/default/files/publications/files/Jain-Health-Insurance-Informal-Economy-India-WIEGO-PB10.pdf.

[R21] Kanore L, Satpute S. 2019. A study of awareness about Ayushyaman Bharat Yojana among low income urban families. An exploratory study. *Remarking an Analisation*. VOL-4*(ISSUE-1* (Part-1)).

[R22] Kansra P. 2015. Socioeconomic determinants of awareness of health insurance among women: an empirical analysis. *IUP Journal of Knowledge Management* 13: 71–83.

[R23] Karpagam S, Vasan A, Seethappa V. 2016. Falling through the gaps: women accessing care under health insurance schemes in Karnataka. *Indian Journal of Gender Studies* 23: 69–86.

[R24] Kieny MP, Bekedam H, Dovlo D et al. 2017. Strengthening health systems for universal health coverage and sustainable development. *Bulletin of the World Health Organization* 95: 537–9. 28670019 10.2471/BLT.16.187476 PMC5487973

[R25] Kirigia JM, Sambo LG, Nganda B et al. 2005. Determinants of health insurance ownership among South African women. *BMC Health Services Research* 5: 1–10. 15733326 10.1186/1472-6963-5-17 PMC553985

[R26] La Forgia G, Nagpal S. 2012. *Government-Sponsored Health Insurance in India*. Washington, D.C: The World Bank.

[R27] Lagomarsino G, Garabrant A, Adyas A et al. 2012a. Moving towards universal health coverage: health insurance reforms in nine developing countries in Africa and Asia. *The Lancet* 380: 933–43. 10.1016/S0140-6736(12)61147-7 22959390

[R28] Lagomarsino G, Garabrant A, Adyas A et al. 2012b. Moving towards universal health coverage: health insurance reforms in nine developing countries in Africa and Asia. *The Lancet* 380: 933–43. 10.1016/S0140-6736(12)61147-7 22959390

[R29] Lu J, Chiang T. 2018. Developing an adequate supply of health services: Taiwan’s path to Universal Health Coverage. *Social Science & Medicine* 198: 7–13. 29272763 10.1016/j.socscimed.2017.12.017

[R30] Moradhvaj, Saikia N. 2019. Gender disparities in health care expenditures and financing strategies (HCFS) for inpatient care in India. *SSM - Population Health* 9: 100372. 10.1016/j.ssmph.2019.100372 PMC6978493 31998823

[R31] Myint C-Y, Pavlova M, Thein KN et al. 2019. A systematic review of the health-financing mechanisms in the Association of Southeast Asian Nations countries and the People’s Republic of China: lessons for the move towards universal health coverage. *PLoS One* 14: e0217278. 10.1371/journal.pone.0217278 PMC6568396 31199815

[R32] Nandi A, Ashok A, Laxminarayan R. 2013. The socioeconomic and institutional determinants of participation in India’s health insurance scheme for the poor. *PLoS One* 8: e66296. 10.1371/journal.pone.0066296 PMC3689788 23805211

[R33] Nandi S, Nundy M, Prasad V et al. 2012. The Implementation of RSBY in Chhattisgarh, India: a study of the Durg district. *Health, Culture and Society* 2: 40–70.

[R34] Nandi S, Schneider H, Dixit P. 2017. Hospital utilization and out of pocket expenditure in public and private sectors under the universal government health insurance scheme in Chhattisgarh state, India: lessons for universal health coverage introduction universal health coverage. *PLoS ONE* 12: e0187904. 10.1371/journal.pone.0187904 PMC5693461 29149181

[R35] National Commission for Backward Classes and Government of India . 1980. Mandal commission: report of the Backword Classes Commission 1980 (Part I and II). Government of India.

[R36] Panda P, Dror IH, Koehlmoos T et al. 2016. Factors affecting uptake of voluntary and community-based health insurance schemes in low- and middle-income countries: a systematic review (no. systematic review 27). London: International Initiative for Impact Evaluation.

[R37] Platteau JP, Ontiveros DU. 2013. Understanding and information failures: lessons from a health microinsurance program in India. *Working Papers 1301*, doi: University of Namur, Department of Economics. 29.

[R38] RamPrakash R, Lingam L. 2021. Why is women’s utilization of a publicly funded health insurance low? a qualitative study in Tamil Nadu, India. *BMC Public Health* 21: 350. 10.1186/s12889-021-10352-4 PMC7881649 33579249

[R39] Rathi P, Mukherji A, Sen G. 2012. Rashtriya Swasthya Bima Yojana: evaluating utilisation, roll-out and perceptions in Amaravati district, Maharashtra. *Economic and Political Weekly* xlvii: 57–64.

[R40] Reddy N, Bahurupi Y, Kishore S et al. 2020. Awareness and readiness of health care workers in implementing Pradhan Mantri Jan Arogya Yojana in a tertiary care hospital at Rishikesh. *Nepal Journal of Epidemiology* 10: 865–70. 32874700 10.3126/nje.v10i2.27941 PMC7423403

[R41] Sen A . 2008. Why and how is health a human right? *The Lancet* 372: 2010. 10.1016/S0140-6736(08)61784-5 19097279

[R42] Shaikh M, Peters SA, Woodward M et al. 2018. Sex differences in utilisation of hospital care in a state-sponsored health insurance programme providing access to free services in South India. *BMJ Global Health* 3: 1–7. 10.1136/bmjgh-2018-000859 PMC6035505 29989065

[R43] Sidibé M. 2016. Universal health coverage: political courage to leave no one behind. *The Lancet Global Health. Elsevier* 4: e355–6. 10.1016/S2214-109X(16)30072-9 27198831

[R44] Smith O, Dong D, Chhabra S. 2019. PM-JAY across India’s states need and utilization PM-JAY policy brief 2. New Delhi: National Health Authority.

[R45] Spaan E, Mathijssen J, Tromp N et al. 2012. The impact of health insurance in Africa and Asia: a systematic review. *Bulletin of the World Health Organization* 90: 685–92. 22984313 10.2471/BLT.12.102301 PMC3442382

[R46] World Health Organization . 2017. Leave no one behind: strengthening health systems for UHC and the SDGs in Africa. Brazzaville: WHO Regional Office for Africa.

